# Nutrigenomics in honey bees: digital gene expression analysis of pollen's nutritive effects on healthy and varroa-parasitized bees

**DOI:** 10.1186/1471-2164-12-496

**Published:** 2011-10-10

**Authors:** Cédric Alaux, Christelle Dantec, Hughes Parrinello, Yves Le Conte

**Affiliations:** 1 INRA, UMR 406 Abeilles et Environnement, Domaine Saint-Paul, 84914 Avignon, France; 2 Institut de Génomique Fonctionnelle, UMR5203 CNRS, U661 INSERM, Universités Montpellier 1 & 2, 34094 Montpellier Cedex 05, France

## Abstract

**Background:**

Malnutrition is a major factor affecting animal health, resistance to disease and survival. In honey bees (*Apis mellifera*), pollen, which is the main dietary source of proteins, amino acids and lipids, is essential to adult bee physiological development while reducing their susceptibility to parasites and pathogens. However, the molecular mechanisms underlying pollen's nutritive impact on honey bee health remained to be determined. For that purpose, we investigated the influence of pollen nutrients on the transcriptome of worker bees parasitized by the mite *Varroa destructor*, known for suppressing immunity and decreasing lifespan. The 4 experimental groups (control bees without a pollen diet, control bees fed with pollen, varroa-parasitized bees without a pollen diet and varroa-parasitized bees fed with pollen) were analyzed by performing a digital gene expression (DGE) analysis on bee abdomens.

**Results:**

Around 36, 000 unique tags were generated per DGE-tag library, which matched about 8, 000 genes (60% of the genes in the honey bee genome). Comparing the transcriptome of bees fed with pollen and sugar and bees restricted to a sugar diet, we found that pollen activates nutrient-sensing and metabolic pathways. In addition, those nutrients had a positive influence on genes affecting longevity and the production of some antimicrobial peptides. However, varroa parasitism caused the development of viral populations and a decrease in metabolism, specifically by inhibiting protein metabolism essential to bee health. This harmful effect was not reversed by pollen intake.

**Conclusions:**

The DGE-tag profiling methods used in this study proved to be a powerful means for analyzing transcriptome variation related to nutrient intake in honey bees. Ultimately, with such an approach, applying genomics tools to nutrition research, nutrigenomics promises to offer a better understanding of how nutrition influences body homeostasis and may help reduce the susceptibility of bees to (less virulent) pathogens.

## Background

The availability of environmental nutrients is a key determinant of organism growth and survival. Therefore, signal transduction and the transcriptional network linking extracellular nutrients to the expression of metabolic genes have been widely studied in model organisms, like single cell organisms [1] and the fruit fly [2]. In addition, nutrients in the diet play a crucial role in developing optimal immune response, such that a deficient or improper diet can have negative consequences on the susceptibility to a variety of pathogens [3,4]. This relationship between health and nutrition has led to the development of a new scientific discipline, called nutrigenomics, that integrates high-throughput genomics tools with nutrition research [5,6]. One of the main goals is to understand how nutrition influences metabolic pathways and homeostasis of the organism and ultimately develop "dietary-intervention strategies to recover normal homeostasis and to prevent diet-related diseases" [6].

The honey bee (*Apis mellifera*) is an important model for such studies, because its dietary requirements are well known [7] and the sequencing of its genome has been completed [8]. If floral nectar (carbohydrates) represents the energetic fuel of honey bees, pollen provides the nutrients required for their internal organ development [7]. Protein constitutes 2.5 to 61% of the dry mass of pollen [9], which is virtually the only source of protein naturally available. Nutrients reported also include lipids (1-20%), amino acids, starch, sterols, vitamins and minerals [10,11]. Together these nutrients combine to make pollen nutrition one of the most important factors influencing the longevity of newly-emerged bees [12]. In addition, proper quantity and quality of pollen not only reduce sensitivity to pesticides [13], but also help the bees to fight against pathogens, like microsporidia [14], bacteria [15] and virus [16], as pollen nutrition actually enhances honey bee immune function [17]. However, the influence of pollen nutrition on honey bee health and resistance to disease cannot be fully understood without determining how nutrients act at the molecular level. This research gap coupled with the availability of new genomic tools in honey bees makes the development of a nutrigenomics approach in honey bees relevant.

Using this approach, we attempted to determine the pollen-induced molecular mechanisms that influence honey bee health. For that purpose, we characterized the transcriptome changes caused by pollen feeding in normal and sick bees. Sick bees were parasitized by the mite *Varroa destructor*, which is an ectoparasite that feeds on the hemolymph of immature and adult bees [18] and in this way infects bees with viruses [19]. The consequences are dramatic since varroa parasitism provokes the deficiency of the honey bee physiological metabolism and immune system [20,21]. Previous studies analyzed the transcriptional response to mite parasitism in prepupae [22] and pupae [23]. Here, the transcriptome analysis was performed on whole abdomen of adult bees, which encompasses the midgut, where pollen digestion takes place, and the fat body, the main site of antimicrobial peptide synthesis and nutrient storage. Here, we used the digital gene expression (DGE) method to perform a deep transcriptome analysis of bees fed with pollen. This method combines the sequencing serial analysis of gene expression (SAGE) principle with the sequencing technology for generating a digital output proportional to the number of transcripts per mRNA [24]. The benefit of not requiring presynthesized oligonucleotide probes (as in microarrays) allows the direct enumeration of transcripts, which is highly replicable, accurate and comparable across experiments [25,26]. In addition, the quantification of weakly expressed genes can be analyzed which cannot be assessed using microarray. After generating the DGE-tag libraries we determined how pollen feeding influences metabolic pathways, genes affecting lifespan, immune functions and virus prevalence.

## Results and discussion

### Statistics of tag sequencing

Four DGE-tag libraries were generated from our experimental groups: control bees without a pollen diet (V-P-), control bees fed with pollen (V-P+), varroa-parasitized bees without a pollen diet (V+P-) and varroa-parasitized bees fed with pollen (V+P+). In total, around 14 × 10^6 ^tags (excluding the adaptor sequences) were sequenced from each library (see Table 1 for details). The libraries from varroa-parasitized bees contained more aligned tags (~7.3 × 10^6 ^tags) than non-parasitized bees (~4.5 × 10^6 ^tags). This higher number of aligned tags is due to the increase in tags matching virus sequences, which reached 1, 417, 481 in the V-P- library, 824, 212 in V-P+, 4, 258, 724 in V+P- and 5, 061, 614 in V+P+. These virus tags represent 32% of the total number of aligned tags in the V-P- library, 18% in V-P+, 60% in V+P- and 67% in V+P+. This clearly shows that varroa triggers a boost of pathogen loads in bees. This was likely induced by repeated varroa feeding on bees hemolymph that not only affect bee physiology but also contribute to the transmission of different viruses [19,21,27,28]. In addition, we noticed that healthy (non-parasitized) bees, which were fed with pollen, had an overall lower load of pathogen tags than bees restricted to a sugar diet. However, few viruses were present at significant different levels between each treatment group (see the *Effects of pollen feeding and varroa parasitism on virus prevalence*).

**Table 1:** Statistics of DGE sequencing

Summary	V-P-	V-P+	V+P-	V+P+
Total tags	20, 397, 219	17, 130, 032	19, 857, 085	20, 633, 649
Total filtered tags	14, 817, 859	13, 508, 333	14, 346, 766	13, 867, 965
Total tags excluding adaptor sequences	14, 471, 228	13, 316, 644	14, 138, 414	13, 598, 573
Total aligned tags	4, 373, 417	4, 616, 713	7, 195, 133	7, 566, 291
Distinct aligned tags	35, 151	38, 719	37, 873	33, 438
Number of unique honey bee gene hit	7, 967	8, 035	8, 142	7, 935

Number of digital tags generated from the four libraries: control bees without a pollen diet (V-P-), control bees fed with pollen (V-P+), varroa-parasitized bees without a pollen diet (V+P-), varroa-parasitized bees fed with pollen (V+P+).

These distinct tags and their genomic count as well as raw data are available from NCBI Gene Expression Omnibus (GEO) database with the accession number: GSE25161. The number of distinct aligned tags was similar between each library (Table 1) and their distribution shows a consistent pattern across the 4 DGE libraries, with most of the distinct tags (34 to 40%) appearing only once and around 40% appearing between two and ten times (Additional file 1). The percentage of tags with a high count number dropped dramatically and only 1% of distinct tags have a count above 1, 000. Such pattern of DGE-Tag profiling was previously observed in honey bee heads [29] and in others insects and vertebrates [30,31]. In total, around 8, 000 honey bee genes were maps from the four abdominal libraries (Table 1), which represents approximately 60% of the genes so far identified from the honey bee genome [8]. As a comparison, 55 to 60% of the genes were also found to be expressed in the bee brain using DGE and microarray analysis [29,32].

### Differentially expressed genes and profiling of marker gene expression

The number of genes, whose expression level was significantly affected by pollen feeding or varroa-parasitism at an adjusted *P*-value for multiple comparison (*Q*-value) <0.01 is given in Table 2 and the lists are provided in Additional file 2. While a similar number of genes were up- and downregulated by pollen nutrition in healthy bees, varroa-parasitism had a major inhibitory effect on gene expression. This inhibition at the transcription level might explain the loss of weight and the decreased metabolism induced by varroa in emerging bees [20].

**Table 2:** Genes differentially transcribed after pollen feeding or varroa parasitism

Comparisons	Upregulated genes	Downregulated genes	Total
V-P+/V-P-	1, 331	1, 140	2, 471
V+P+/V+P-	146	1, 677	1, 823
V+P-/V-P-	169	2, 474	2, 643
V+P+/V-P+	25	3, 789	3, 814

Genes were considered significantly differentially expressed between two treatments if their associated *Q*-value < 0.01.

**Figure 1:**
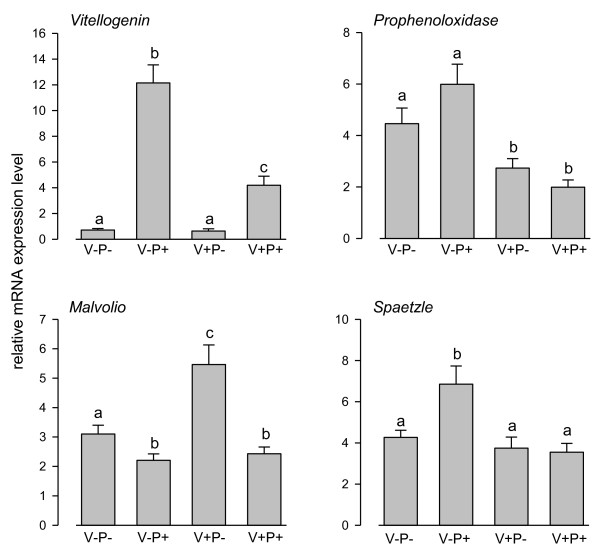
**Marker genes affected by pollen feeding or varroa parasitism**. Expression values of the 4 genes chosen among the set of genes differentially transcribed between the different treatments. RT-PCR data normalized to *β-actin *are shown. Means ± SE are shown for 8 pools of 3 bees per treatment. Different letters indicate significant differences detected by Mann-Whitney U tests.

To assess the reliability of our DGE-tag profiling, we checked whether the regulation of some candidates genes was consistent with the pattern of expression previously reported in earlier studies and performed reverse-transcriptase PCR (RT-PCR) on those genes. Vitellogenin (Vg) is a yolk protein taken up by developing oocytes and associated to egg production in queens [33,34] but also has antioxidant functions that protect bees from oxidative stress and enhance longevity [35,36]. Therefore, *Vg *level can be assimilated to an indicator of longevity. Since, pollen nutrition promotes the development of fat body [12,17] and *Vg *is synthesized in the fat body [35], we expected an increase in *Vg *level in bees fed with pollen as previously found [37]. That was both confirmed by the DGE and RT-PCR analysis (Additional file 2 and Figure 1). This suggests that one way by which pollen feeding increase longevity [7] is via the increase of *Vg *level. Interestingly, varroa parasitism decreased the *Vg *level in bees fed with pollen (see also [38]), but this level was still significantly higher than in parasitized bees restricted to a sugar diet.

Another interesting gene is *malvolio *(*mvl*), which encodes a manganese transmembrane transporter involved in sucrose responsiveness [39]. In honey bees, foraging workers that are more responsive to sucrose have a higher level of *mvl *mRNA in the brain than young nurses taking care of the brood inside the hive [40]. Pollen feeding induced an expression pattern opposite to *Vg *(Figure 1). Bees fed only with sugar had a higher level of *mvl *in the abdomen. Since old foragers feed mainly on carbohydrates and young nurses feed on both carbohydrates and pollen [41], emerging bees that are restricted to a sugar diet tend to develop a *mvl *expression profile similar to foragers as compared to bees fed with pollen, which are more similar to nurse bees. Indeed, as opposed to poor diet, a high protein diet might stimulate nurse tasks via the development of hypopharyngeal glands used for secreting larval food [7]. Similarly, *v*arroa parasitism induced an increase in *mvl *level (although not significant in bees fed with pollen), suggesting also a forager profile.

The expression level of the gene *prophenoloxidase *(*PPO*), involved in insect immunity [42], was also confirmed by RT-PCRs (Figure 1) and previous data. Indeed, varroa parasitism inhibited *PPO *expression, which was consistent with the immune suppression previously reported [21], but pollen feeding did not affect *PPO *level (see also [17] at the enzyme activity level).

Finally, we checked the expression level of the *Drosophila *ortholog *spaetzle*, which is a member of the *Toll *immune signaling pathway against fungi and bacteria [42]. The DGE-tag profiling of this gene, confirmed by RT-PCR, showed that pollen feeding significantly increased the number of *spaetzle *transcripts in healthy bees (Figure 1), which suggests that pollen nutrients can enhance immune function in bees. The expression of this gene was reduced by varroa parasitism in bees fed with pollen, supporting again the immune suppression induced by varroa. Altogether, these results validated the DGE-tag profiling of the four libraries.

### Functional analysis of differentially expressed genes

**Figure 2:**
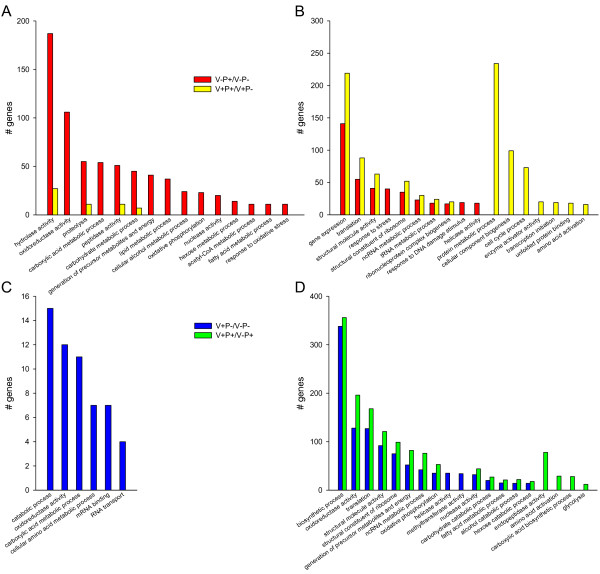
**Functional (Gene Ontology) analysis of genes affected by pollen feeding (A, B) or varroa parasitism (C, D)**. The lists of genes regulated by pollen feeding or varroa parasitism were analyzed for statistical enrichment of associated GO terms (*P *< 0.05), relative to the representation of these terms for all genes analyzed with DGE. Molecular function and biological process that were enriched in upregulated (A, C) and downregulated gene sets (B, D) are shown.

Gene Ontology analysis was performed to explore which functional components are regulated by pollen feeding and affected by varroa parasitism. Although the pollen wall, composed of sporopollenin and cellulose, is poorly digested by honey bee digestive enzymes, 50 to 98% of pollen contents can be extracted from ingested pollen, depending on pollen type and age of the bee [10]. Bees of 9 days-old, close to the age of bees that we analyzed, can digest as much as 95% of pollen contents [41]. The upregulation of many metabolic functions in bees fed with pollen highlights the high digestibility of pollen (Figure 2A). Indeed, in response to pollen nutrients, composed mostly of proteins and lipids, genes involved in proteolysis, lipid metabolism and coding for peptidases were upregulated. Carbohydrate metabolism was also upregulated by pollen feeding, suggesting a digestive response to pollen starches. Besides the metabolic categories, "oxidative phosphorylation" and "generation of precursor metabolites and energy" showed significant enrichment among the tested categories. This might not be surprising since nutrients are used to produce energy, like ATP via oxidative phosphorylation.

Groups of genes implicated in translational processes and ribosome biogenesis were downregulated by pollen feeding (Figure 2B). In all eukaryotes, ribosome biogenesis and translation regulation are essential for tissue growth and are regulated by food intake. Proper regulation of those processes are required to ensure cell and tissue homeostasis upon changes in nutrition and are inhibited during starvation for dietary nutrients [2]. Therefore, an upregulation of ribosome biogenesis and translation was expected upon pollen feeding. However, the majority of pollen consumption and tissue development (e.g. hypopharyngeal glands, ovaries and fat body) occur within the first week after adult emergence [43]. The prior completion of physiological development before our analysis could explain the downregulation of translational processes and ribosome biogenesis.

The effect of pollen nutrition on honey bee physiology was dramatically altered by varroa parasitism. Whereas groups of genes involved in ribosome biogenesis and translation were also downregulated, metabolic categories were no longer upregulated (Figure 2A). Only small sets of genes involved in proteolysis, peptidase activity and carbohydrates metabolism were still upregulated by pollen nutrition. In contrast most of the genes implicated in protein metabolism were downregulated in varroa-parasitized bees (Figure 2B), demonstrating that varroa mites inhibited the digestion and/or the use of proteins. In addition, the "lipid metabolic process", "oxidative phosphorylation" and "generation of precursor metabolites and energy" categories were no longer enriched. Metabolic processes were also found to be mostly downregulated by varroa parasitism in prepupae [22]. Altogether, these results indicate that parasitized bees could not correctly assimilate and use the pollen nutrients required for their physiological development.

This was further confirmed by the effect of varroa on the bee transcriptome. Functional analysis showed that all of the previous categories, such as ribosome biogenesis, translation, metabolic functions and energy production, were inhibited by the varroa mite (Figure 2D). The inhibition by varroa of ribosome biogenesis and translation, essential to cell growth, might lead to lower tissue development. The lower concentrations of proteins and carbohydrates induced by varroa [20] might be explained by our results on the inhibition of metabolic genes and genes coding for endopeptidase. This reduction of metabolic reserves induced by varroa was reported at the emergence [20], therefore the downregulation of metabolic genes lasting 8 days after the emergence in bees fed with pollen, demonstrates that pollen nutrients are not able to compensate those losses.

A large set of genes were regulated by pollen feeding both in parasitized and non-parasitized bees and presented a similar expression pattern (Table 3 part 1). However, a subset of genes was upregulated by pollen feeding in healthy bees and downregulated in parasitized bees. A similar pattern was observed regarding the effect of varroa on bees fed with or without pollen (Table 3 part 2). In order to decipher the biological meaning of those opposite regulations, we performed a functional analysis of those subsets. Among the subset upregulated by pollen feeding in healthy bees but downregulated in parasitized bees, groups of genes are involved in ribosome biogenesis, translation, protein metabolism and energy production (Additional file 3 part A). The subset upregulated by varroa parasitism in bees fed without pollen but downregulated in pollen-fed bees, showed significant enrichment for genes coding for oxidoreductase and involved in amino acid metabolism (Additional file 3 part B). Those results confirmed the negative effect of varroa on those previously reported functions. Indeed, the energy metabolism and nutrient demand of the mite is high, utilizing up to 25% of the nutritional reserves of pupae [44]. The decrease in metabolic reserves lasting in adults might come from the development of viruses transmitted by mites at the larvae or pupae stage.

**Table 3:** Overlap of genes regulated in the abdomen by pollen feeding or varroa parasitism

**1)**		**Varroa -**			**2)**	**Pollen -**			
			
		**Pollen ↑**	**Pollen ↓**	**Significance**			**Varroa ↑**	**Varroa ↓**	**Significance**
			
Varroa +	Pollen ↑	111	8	*χ2 *= 190.1	Pollen +	Varroa ↑	8	7	*χ2 *= 74.6
	Pollen ↓	244	624	*P *< 0.001		Varroa ↓	81	1925	*P *< 0.001

1) Number of genes regulated by pollen feeding in bees parasitized (Varroa +) or not by varroa (Varroa -). 2) Number of genes regulated by varroa parasitism in bees fed with (Pollen +) or without pollen (Pollen -). ↑ and ↓ indicate genes that are up- or downregulated. Chi-square tests with Yates correction were performed to determine whether overlapping genes follow the same pattern of expression between the two conditions.

Similar results to the functional analysis were found with the pathways analysis (Additional file 4). Using the later analysis, we also studied the effect of pollen and varroa on amino acids. Indeed, honey bees require ten essential amino acids for their adult development: arginine, histidine, lysine, tryptophan, phenylalanine, methionine, threonine, leucine, isoleucine, and valine [45]. This means those amino acids cannot be synthesized *de novo *by the bees and therefore must be supplied directly in the diet either as free amino acids or digested from proteins. We therefore looked at the amino acid metabolism pathways that were significantly enriched after pollen feeding and found that four of them (arginine, leucine, isoleucine, and valine) were upregulated (Additional file 4). Interestingly, five of those amino acid pathways were downregulated by varroa (lysine, tryptophan, leucine, isoleucine, and valine), confirming that varroa significantly reduces the development of bees. However, none of these essential amino acid pathways were significantly affected in varroa-parasitized bees fed with pollen compared to parasitized bees restricted to a sugar diet, suggesting that pollen nutrients compensated for the negative impacts of varroa on the metabolism of those amino acids.

### Effects of pollen feeding and varroa parasitism on the nutrient sensing pathway

**Figure 3:**
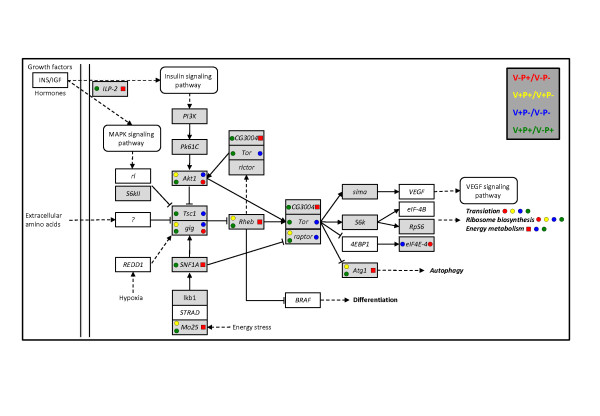
**mTOR signaling pathway affected by pollen feeding or varroa parasitism**. Genes from the *Drosophila *mTOR signaling pathway that were differentially transcribed after pollen feeding or varroa parasitism. The pathway was modified from KEGG: Kyoto Encyclopedia of Genes and Genomes http://www.kegg.com/. Grey indicates the presence of genes in the bee genome. Squares and circles indicate genes and enriched functions that were up- and downregulated between two treatments, respectively.

The insulin/TOR pathway is a conserved signaling cascade that functions as a nutrient sensing pathway by linking food-intake to animal growth and metabolism, including reproduction and lifespan [46,47]. In honey bees, this pathway plays a major role in the regulation of aging of individuals [35,48] but also at a social level by regulating the division of labor between worker bees [49,50]. Less is known about the nutrient regulation of this pathway in honey bees. However, a recent study showed that the inhibition of *Insulin receptor substrate *expression in peripheral tissues (as opposed to central brain tissues) made forager bees collect pollen rather than nectar [51]. By analyzing the DGE-tag profiling of peripheral tissues (abdomen) we found that most of the genes from the mTOR signaling pathways were upregulated by pollen nutrition in healthy bees (Figure 3). The *insulin-like peptide 2 *(*ILP-2*) expression notably increased in response to pollen nutrition, as it was found in response to amino acids [48], which might promote nurse physiology that extends bee longevity [52]. Altogether these results show that the insulin/TOR pathway responds positively to pollen nutrition and might trigger the pollen-induced physiological development. The opposite effect was observed regarding *ILP-1*, with a higher expression under poor nutrition conditions [50]. However, those inverse patterns might actually be explained by the functional differences of *ILP-1 *and *ILP-2 *[52] . In varroa-parasitized bees, pollen nutrition tended to inhibit this pathway. This could be caused by the higher prevalence of viruses in parasitized bees fed with pollen compared to parasitized bees fed only with sugar (see *Effects of pollen feeding and varroa parasitism on virus prevalence*). Indeed, varroa parasitism (coupled with a higher viral load) induced a similar pattern of the TOR pathway (Figure 3). The parasite inhibits protein metabolism and decreases the nutrient reserve of bees (see above), which might be explained by the downregulation of the TOR pathway mediating tissue growth in response to nutrient intake. This is further confirmed by the inhibition of *slimfast *by varroa parasitism in bees fed with pollen. The downregulation of this gene, which encodes an amino acid transporter and is a downstream target of TOR pathway, causes a deficient global growth in *Drosophila *similar to that seen in individuals raised under poor nutritional conditions [46,53].

TOR is known to respond to the presence of amino acids and induces an upregulation of ribosome biogenesis, translation [2,54-57] and energy metabolism [58,59] required for tissue growth. Interestingly, the downregulation of TOR pathway was followed by the downregulation of each biological process (Figure 3), indicating that the same mechanisms of the nutrient sensing pathway are found in honey bees. The upregulation of the pathway in pollen-fed bees was followed by an increase in energy metabolism but the ribosome biogenesis and translation were both inhibited. As explained above, it is possible that tissue development that requires the activation of translational processes was already completed.

The expression level of genes did not always match well to the activator/inhibitor functions established in the *Drosophila *TOR pathway. The expression patterns correspond to global DGE-tag profilings of whole bee abdomens but many genes possess tissue-specific functions. Therefore, targeting specific tissues like the fat body, which has important storage functions associated with nutrition, should provide more accurate expression data for pathway analysis.

### Effects of pollen feeding and varroa parasitism on genes affecting lifespan and immune genes

**Figure 4:**
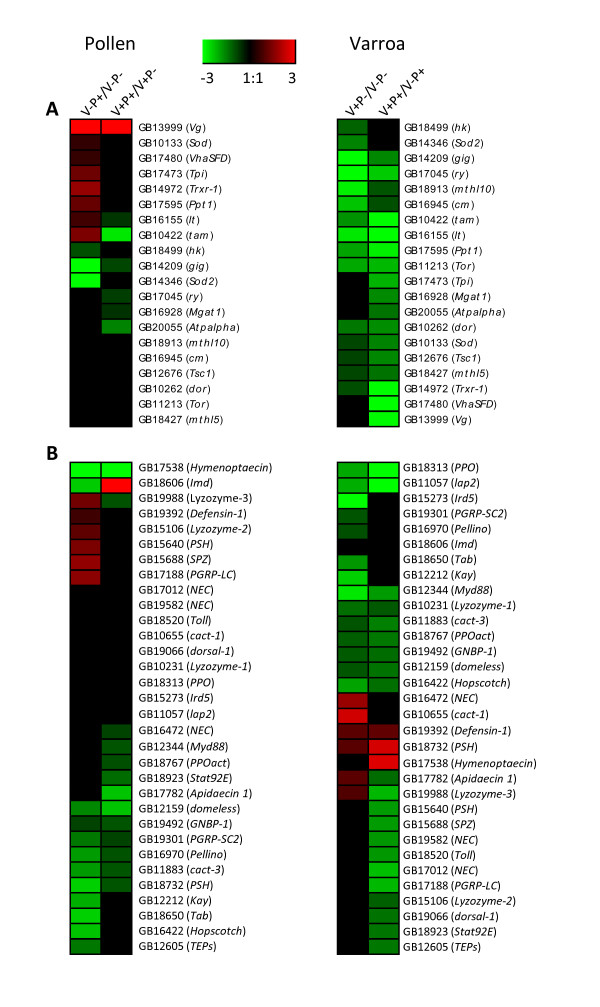
**Immune and lifespan genes affected by pollen feeding or varroa parasitism**. A) Genes from our gene sets that are involved in lifespan in *Drosophila *and honey bees (i.e. *Vg*). B) Immune genes found in honey bees (the list of genes originated from [42]). Colors scale from the heatmap (green to red) indicates log2 transcription ratios. For each gene, accession number and annotation are indicated.

Since pollen nutrition and varroa increases and reduces the longevity of bees [12,60], respectively, we looked at the expression pattern of genes that have been found to affect longevity in *Drosophila*. Those genes were found by using the functional analysis from DAVID 6.7 bioinformatic resources [61] and GoToolbox [62]. We found 20 genes including *Vg*, known to affect longevity, to be differentially expressed between the different treatments. A mutation at the loci of each of those genes causes a decrease in longevity except for *Tor*, whose inhibition increase longevity [63]. Among those genes, we expected to find a larger set to be upregulated by pollen feeding than downregulated and inversely regarding varroa effect. According to our expectation, 7 out of 10 genes regulated by pollen intake were upregulated in non-parasitized bees (Figure 4A). For example, *Sod *and *Trxr-1*, which encodes a cytoplasmic Cu-Zn superoxide dismutase and a thioredoxin reductase, respectively [64], are known to increase lifespan [65,66], thanks to the degradation of superoxide radicals and H_2_O_2 _or the neutralization of reactive oxygen species. The positive effect of pollen feeding on the expression pattern of both genes was confirmed by reverse-transcriptase PCR (RT-PCR) analysis performed on a different set of experimental bees (n = 8 pools of bees per treatment, Additional file 5). Those genes might be involved in the pollen-induced molecular mechanisms that enhance life expectancy of honey bees. However, *Sod 2 *(mitochondrial Mn superoxide dismutase), which is also involved in degradation of superoxide radicals and H_2_O_2 _[64], was not affected by pollen feeding (Additional file 5), contrary to the downregulation found with the DGE analysis. Due to the higher level of Vg (Figure 1), a longer lifespan is expected in parasitized bees supplied with pollen compared to parasitized bees fed only with sugar (Figure 4A). However, except *Vg*, genes that were regulated by pollen intake were all downregulated. Whether pollen nutrition can affect varroa resistance remains to be tested but it might be possible that the effect of pollen on the genes affecting longevity is compensated by the increase in Vg. The picture was clearer with regards to the effect of varroa on the 19 genes affecting longevity (Figure 4A and Additional file 5). All of them were downregulated (including *Sod 2 *in presence of pollen, Additional file 5) supporting a decrease of longevity induced by varroa.

Next, we examined the effect of pollen intake and varroa on the expression pattern of immune genes, that have been previously annotated [42]. Few immune genes were upregulated by pollen feeding in healthy bees (Figure 4B). Three of them encoded antimicrobial peptides (AMPs) (*Lyzozyme-2 *and *-3, Defensin-1*), which might be linked to a higher content of fat body, the major site of synthesis of AMPs. Interestingly, in the absence of immune challenge (healthy bees), *Spaetzle *and the gene coding for the peptidoglycan recognition protein (*PGRP*)-*LC*, which are activators of the *Toll *and *Imd *pathways respectively, were both upregulated by protein feeding. The upregulation of those key immune genes confirmed by RT-PCR analysis (Figure 1 for *Spaetzle *and Additional file 5 for *Defensin-1 *and *PGRP*-*LC*) suggests that bees might be able to mount an efficient immune response to pathogen infections. In parasitized bees, only *Imd*, a specific gene for antimicrobial defense, was upregulated by pollen feeding. A slight increase of *Imd *expression was also observed with RT-PCR analysis although not significant (p = 0.058, Additional file 5). In healthy bees, *Imd *expression did not change after pollen feeding (Additional file 5). Since, protein and amino acid nutrition can enhance immune functions in humans [4,67] and insects [68,69], an upregulation of immune genes was expected. The functional analysis showed that in varroa-parasitized bees fed with pollen, a significant number of genes involved in protein metabolism were downregulated compared to parasitized bees fed only with sugar (Figure 2B). A deficiency in protein metabolism might have affected the immune system of those bees. This immunosupression could be explained by the higher virus load in parasitized bees fed with pollen compared to parasitized bees fed only with sugar (see *Effects of pollen feeding and varroa parasitism on virus prevalence*). However, when fed with pollen, it is possible that parasitized bees invest more energy in other defense mechanisms than immunity which is inhibited by varroa, e.g. the antioxidant system (higher *Vg *level in bees fed with pollen).

Varroa induced the inhibition of most of the immune genes in bees fed with or without pollen (including *PGRP*-*LC*, Additional file 5). However, a few immune genes were upregulated, like the AMPs (*Defensin-1, Hymenoptaecin, Apidaecin-1 *and *Lyzozyme-3*) and *cact-1*, which controls the production on AMPs [70]. The upregulation of *Defensin-1 *by varroa parasitism was confirmed by RT-PCR analysis (Additional file 5). Those genes, except *Lyzozyme-3 *have already been found to be upregulated by a immune challenge [42]. The mechanisms triggering the immunosupression are still misunderstood but one explanation might be that the deficient protein metabolism induced by varroa and associated viruses (see above) block the development of immune function.

Immune genes that were upregulated by pollen feeding and varroa (Figure 4B) belonged to the antibacterial peptide family or were key activators of immune pathways. Since the characterization of immune defense in honey bee is still at his infancy, it is possible that the others immune genes don't actually play a major role in immune response in bees. Indeed, genes from immune signaling pathway have usually different roles, like developmental functions.

### Effects of pollen feeding and varroa parasitism on virus prevalence

Using the DGE-tag libraries from honey bee abdomen samples, we performed a metagenomic analysis to determine the effects of pollen feeding and varroa on the prevalence of 10 honey bee viruses: Chronic bee paralysis virus RNA 1, Chronic bee paralysis virus RNA 2, Sacbrood virus, Deformed wing virus, Black queen cell virus, Acute bee paralysis virus, Kashmir bee virus, Kakugo virus, *Varroa destructor *virus 1 and Israel acute paralysis virus. All of them were identified in our DGE-tag libraries but very low numbers of tag copies of the Chronic bee paralysis virus RNA 1, Chronic bee paralysis virus RNA 2, Sacbrood virus, Black queen cell virus, Acute bee paralysis virus, Kashmir bee virus and Israel acute paralysis virus were found (from 1 to 65, see data deposited in GEO database: GSE25161) and no difference in their abundance was detected between treatments. However, tags mapping the deformed wing virus (DWV), *Varroa destructor *virus (VDV) and Kakugo virus (KV) were highly prevalent and significantly affected by the different treatments (Table 4). *Varroa destructor *is an efficient vector of bee viruses by promoting their transmission and development (DWV [19,21], VDV [27], KV [28]) but the presence of viruses in control bees can be explained by a vertical transmission from queens and drones to worker offspring through fertilized eggs [71].

**Table 4:** Effects of pollen feeding and varroa parasitism on the prevalence of honey bee virus

		Number of tag copies				Tag odds ratio		
**Tag ID**	**V-P-**	**V-P+**	**V+P-**	**V+P+**	**V-P+/V-P-**	**V+P+/V+P-**	**V+P-/V-P-**	**V+P+/V-P+**

Deformed winged virus-9489	191, 776	533, 740	1, 653, 968	2, 400, 089	2.85	ns	6.51	3.55
Deformed winged virus-4761	154	254	4, 110	10, 825	1.56	2.51	16.23	26.04

*Varroa destructor *virus-5865	676	902	4, 778	10, 075	ns	2.01	4.3	6.82
*Varroa destructor *virus-9462	10, 611	3, 721	39, 324	31, 578	0.33	ns	2.26	5.2
*Varroa destructor *virus-9753	1, 202, 745	277, 601	2, 514, 691	2, 556, 286	0.17	ns	ns	7.98

Kakugo virus-9501	94	245	1, 139	8, 695	2.47	7.27	7.37	21.68

Virus tags that were differentially expressed between two conditions (*Q*-value < 0.01) in pools of 12 bees. Numbers of tag copies and odds ratio are indicated. *ns *stands for "not significant". Each tag ID is composed of the corresponding virus name and tag position in the sequence (indicated by the last numbers).

As expected, varroa provoked a boost of virus development (Table 4). Since AMPs have a broad-spectrum antimicrobial activity against bacteria, yeast, fungi, parasites but also viruses [72], the upregulation of AMPs in varroa-parasitized bees (Figure 4B) might represent the immune response to the high viral infection. Different studies showed that pollen nutrients can help the bees to fight against parasites and pathogens [14,15]. For example, a recent study found that pollen nutrition reduce the prevalence of DWV in normal adult bees [16]. We obtained similar results in healthy (non-parasitized) bees with pollen nutrition reducing the load of VDV. However, we observed the inverse pattern with DWV and KV. Viruses utilize cell machinery to multiply, so higher number of cells available could contribute also to a better development of viral population. For example, DWV has been found to multiply in the bee fat body [73] and this tissue is more developed in pollen-fed bees. We also found that pollen feeding increased viruses prevalence in varroa-parasitized bees, indicating pollen nutrition is not effective for limiting virus loads in heavily infected bees. Larger physiological machinery might, to the contrary, favor their multiplication, but it might also help bees to withstand viral infection.

## Conclusions

With this study, we showed that nutrigenomics, originally developed for human health purposes, is also promising for deciphering the nutritive influence on metabolic pathways in insects. More specifically, this work is the first step towards understanding how pollen nutrients shapes honey bee health. While pollen nutrition enhances macromolecule metabolism and activates the TOR pathway required for the tissue growth and development, it also contains nutrients that stimulate the expression of genes involved in longevity, like genes coding for antioxidants (*vitellogenin *and *superoxide dismutase*). However, the negative impacts of varroa on the bee metabolism and immune functions could not be reversed by pollen feeding. By inhibiting the macromolecule metabolism (notably proteins), varroa parasitism prevented bees from accessing the beneficial effects of pollen. This demonstrates that pathology associated to varroa is extremely virulent and difficult to reverse, probably due to the multiplication of multiple viruses transmitted by the mite.

In the future, studying more specific tissues (like fat body) and particular dietary components will hopefully provide a more complete and comprehensible picture of the beneficial or adversarial effects of pollen components in bees. Finally, given that honey bees are victims of a wide range of pathogens that might be less virulent than varroa, developing dietary intervention based on knowledge of nutritional requirement and status can be useful to prevent, mitigate or cure chronic disease.

## Methods

### Experimental treatments

The experiment was performed with local hybrid honey bees (*A. m. ligustica*/*A. m. mellifera*). In order to obtain a large number of varroa-infected bees, an infested colony that was not treated with miticide was selected. The multiple-mated queen was placed in a queen cage to stop egg-laying for 10 days. After 10 days, all the brood produced before the queen confinement was sealed and the mites had no brood cell to parasitize. Then, the queen was transferred to a queen-excluder for 3 days with an empty frame. The brood produced by the queen on this frame was then uniformly parasitized by varroa mites. Three weeks after the queen laid eggs in the queen-excluder, the frame was removed from the colony and the sealed cells were opened carefully to remove the adult workers just before emergence and to check for the presence of reproducing varroa mites. Varroa-parasitized bees with deformed wings were discarded due to their extremely short lifespan.

Varroa-parasitized and non-parasitized bees (control) were placed in distinct cages in the dark at 32°C and 70% RH and fed *ad libitum *with candy (30% honey, 70% powdered sugar) and water. To simulate as much as possible the colony rearing conditions, cages were exposed to a Beeboost^® ^(Pherotech), releasing one queen-equivalent of queen mandibular pheromone per day. Half of the groups were also fed with a polyfloral pollen diet, which was replaced every day and prepared with fresh pollen obtained from our colonies in Avignon (France) and 1/10 water. Thus we obtained 4 experimental groups: control bees without a pollen diet (V-P-), control bees fed with pollen (V-P+), varroa-parasitized bees without a pollen diet (V+P-) and varroa-parasitized bees fed with pollen (V+P+). Since their pollen consumption diminishes when bees are 8-10 days old [41], bees from each group were flash frozen in liquid nitrogen at the age of 8 days. One group (pool of 12 bees) per treatment was used for comparing the 'average' responses to the different treatments with the digital transcriptome analysis. Single replicate analyses have generated consistent results in previous studies [30,31,74], but confirmation of some of the results obtained from digital expression analysis was performed with RT-PCR on four new experimental replicates (see below). The quantities of pollen consumed in the V-P+ and V+P+ groups were similar (8.2 ± 2 and 7.7 ± 2.6 mg per bee and per day, respectively).

### Digital transcriptomics

Twelve abdomens per group were homogenized in Trizol (Invitrogen cat. No. 15596-026) and RNA extraction was carried out as indicated in the Qiagen RNeasy kit (Qiagen cat. No. 74104) for total RNA with on-column DNase I treatment (Qiagen cat. No. 79254).

Sequencing libraries were constructed using Illumina's DGE Tag Profiling DpnII Sample Prep kit (Illumina cat. No. FC-102-1007) according to the manufacturer's instructions (cat. No. 1004241 rev.A). Briefly, polyA+ RNA were isolated from 2 μg of total RNA using Sera-magnetic oligo-dT beads. Reverse transcription was performed on immobilized polyA+ RNA using SuperScript II Reverse Transcriptase (Invitrogen cat. No. 18064-14). Second strand was synthesized using DNA Polymerase I. Immobilized cDNAs were digested with DpnII. Digested fragments were washed off, and the retained cDNA fragments were ligated to GEX DpnII Adapter 1. Tags were released by MmeI digestion generating 20 bp tags that are dephosphorylated using CIAP and ligated to GEX Adapter 2. The adapter-ligated cDNA tags were enriched using 15 cycles of PCR, and purified on a 6% Novex TBE PAGE gel (Invitrogen cat. No. EC6265Box). 85-bp fragments were excised from the gel, eluted and precipitated. Tags were quantified on an Agilent Bioanalyzer using DNA1000 chip (Agilent cat. No. 5067-1504). Each library was diluted to 10 nM, denatured, and diluted again to 8 pM. 100 μl of the diluted library were hybridized on a lane of an Illumina's Flow Cell. Clustering and 36 cycles sequencing were performed according to Illumina's instructions.

### Analysis and mapping of DGE tags

We first filtered the raw data via the default Illumina pipeline quality filter, which calculates a Chastity score as the ratio of the highest of the four (base type) intensities to the sum of highest two. This score is used to remove clusters with low signal to noise ratio (often caused by clusters being too close to each other so their signals bleed into one another). The sequences were passed through the chastity filter of value > = 0.6. Then, adapters were trimmed from tag sequences by an in-house script. Reads displaying sequence ambiguity were excluded from further analysis. For each library, read counts were recorded for every unique sequence. Tag sequences were mapped to the honey bee genome (BeeBase Genome - release 2 http://genomes.arc.georgetown.edu/drupal/beebase/?q=home) with Eland v1.4, and only perfect tag matches of coding sequences were kept for statistical analysis. Mapping was also performed on different sequences of honey bee virus genomes (Chronic bee paralysis virus RNA 1: GenBank EU122229, Chronic bee paralysis virus RNA 2: EU122230, Sacbrood virus: AF092924, Deformed wing virus: AJ489744, Black queen cell virus: AF183905, Acute bee paralysis virus: AF150629, Kashmir bee virus: AY275710, Kakugo virus: AB070959, Varroa destructor virus 1: AY251269 and Israel acute paralysis virus: EF219380).

In the case of tag census, the probability distribution of the measured parameter 'tag abundance' is known *a priori *and follows a hypergeometric distribution. Accordingly, the probability that 2 census of the same transcript in 2 different conditions are significantly different can be calculated without replicate. Differentially expressed tags were therefore identified by a Fisher's exact test with Benjamini-Hochberg method, which controls the expected proportion of incorrectly rejected null hypotheses (type I errors), to compute the False Discovery Rate and a cut-off *Q*-value of 0.01 as implemented in the SAGE Genie resource http://cgap.nci.nih.gov/SAGE. The expression pattern of the tags was based on their sequence odds ratio between two pools A and B: (sequences in A/total sequences in pool A)/(sequences in B/total sequences in pool B).

### Functional analysis

We explored whether any particular molecular functions or biological processes from the differentially expressed gene lists were represented by larger numbers of genes than expected on the basis of chance. *Drosophila melanogaster *orthologs were identified by reciprocal best BLASTX match to bee genes, and Gene Ontology (GO) terms were assigned based on annotation of *Drosophila *genes. GOToolBox [62] was used to identify overrepresented terms (hypergeometric tests followed by the Benjamini Hochberg correction for multiple testing). Enrichment analysis of bio-pathways were performed using DAVID 6.7 bioinformatic resources [61].

### Verification by reverse-transcriptase PCR

RT-PCR was performed on selected genes known to be affected by nutrition and varroa parasitism (*vitellogenin *(*Vg*), *malvolio *(*mvl*), *Prophenoloxidase *(*PPO*) and *Spaetzle *(*spz*)), three genes involved in lifespan (*Superoxide dismutase *(*Sod*), *Superoxide dismutase 2 *(*Sod2*), *Thioredoxin reductase 1*(*Trxr-1*)) and three immune genes (*Peptidoglycan recognition protein LC *(*PGRP-LC*), *defensin1, Immune deficiency *(*Imd*)). The RT-PCR analysis was carried out on a different set of bees obtained by using the same experimental procedure (see above). Four experimental replicates per treatment were performed and 2 pools of 3 bees per replicate (cage) were analyzed (n = 8 pools/treatment). The transcript abundance was measured for with an M × 3000 P QPCR Systems (Agilent) and the SYBR green detection method (Agilent cat. No. 600828). RT-PCR values of the selected genes were normalized by using an exogenous human RNA control (*β-actin*) spiked during cDNA synthesis performed with the SuperScript III (Invitrogen, France). Relative expression was calculated by raising 2 to the power of the difference in Ct values. Primer sequences are listed in Additional file 6.

## Authors' contributions

CA and YLC conceived and designed the experiments. CA performed the experiments. CA and HP carried out the molecular genetic studies. CA and CD analyzed the data. CA and YLC wrote the manuscript. All authors read and approved the final manuscript.

## Supplementary Material

Additional file 1 **Frequency of distinct aligned tags in the four DGE libraries**. Click here for file

Additional file 2 **Lists of tags differentially expressed after pollen feeding or varroa parasitism**. The number of copies found in each library, the sequence odds ratio and the *Q*-value are given for each tag. Corresponding honey bee gene and *Drosophila *ortholog are shown. Each tag ID is composed of the corresponding honey bee gene name and tag position in the gene (indicated by the last numbers). *NaN *stands for "not a number" and occurs when the denominator of the odds ratio is 0 (i.e., there are no sequences of a gene in the second tag library). Click here for file

Additional file 3 **Molecular function and biological process that were enriched in overlapping gene sets**. A) Molecular function and biological process from genes that were upregulated in V-P+ but downregulated in V+P+. B) Molecular function and biological process from genes that were upregulated in V+P- but downregulated in V+P+. The number of genes differentially expressed in each pathway is shown. Click here for file

Additional file 4 **Molecular pathways affected by pollen feeding or varroa parasitism**. Pathways that were significantly enriched (*P *< 0.05) in the different gene sets are shown. The analysis was done with DAVID 6.7 bioinformatic resources. Click here for file

Additional file 5 **Analysis with RT-PCR of selected immune and lifespan genes affected by pollen feeding or varroa parasitism**. Expression values of selected lifespan (*Sod, Sod2, Trxr-1*) and immune genes (*PGRP-LC, defensin1, Imd*) differentially transcribed between the different treatments. RT-PCR data normalized to *β-actin *are shown. Means ± SE are shown for 8 pools of 3 bees per treatment. Different letters indicate significant differences detected by Mann-Whitney U tests. Click here for file

Additional file 6 **Sequences of qPCR primers**. Click here for file
