# Supplementary figures and images for: Nutrigenomics in honey bees: digital gene expression analysis of pollen's nutritive effects on healthy and varroa-parasitized bees
Source: BMC Genomics. 2011 Oct 10;12:496. doi: 10.1186/1471-2164-12-496 (PMC3209670; doi:10.1186/1471-2164-12-496)

V-P-

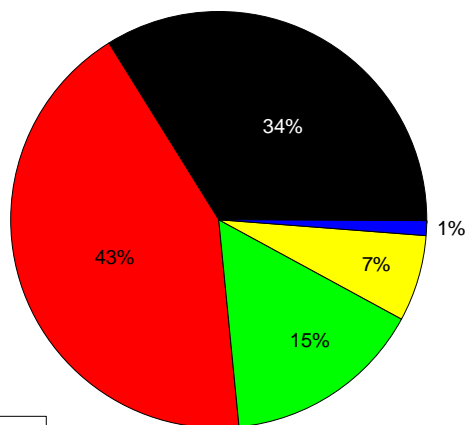

V-P+

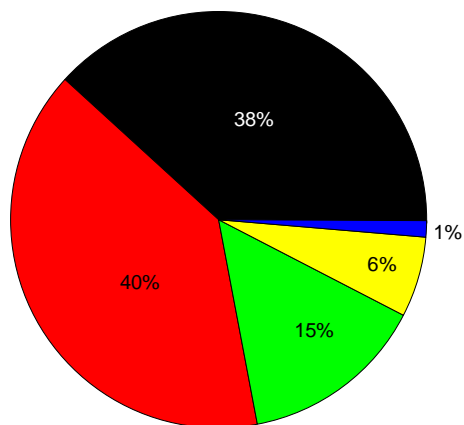

V+P-

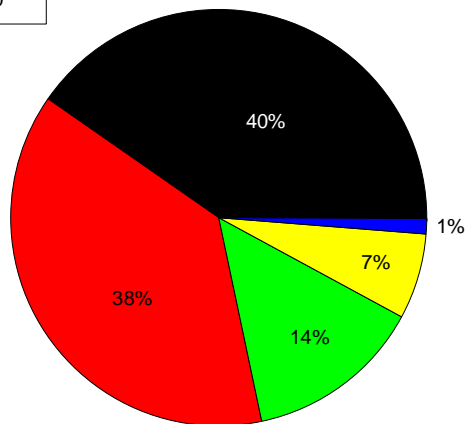

V+P+

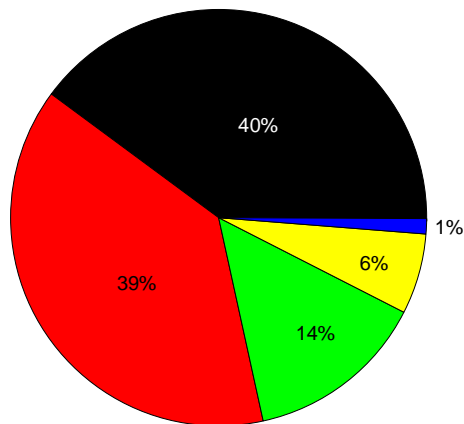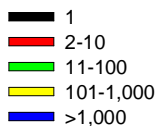

Supplement: Additional file 1 — Frequency of distinct aligned tags in the four DGE libraries. [file 1471-2164-12-496-S1.PDF]

**A**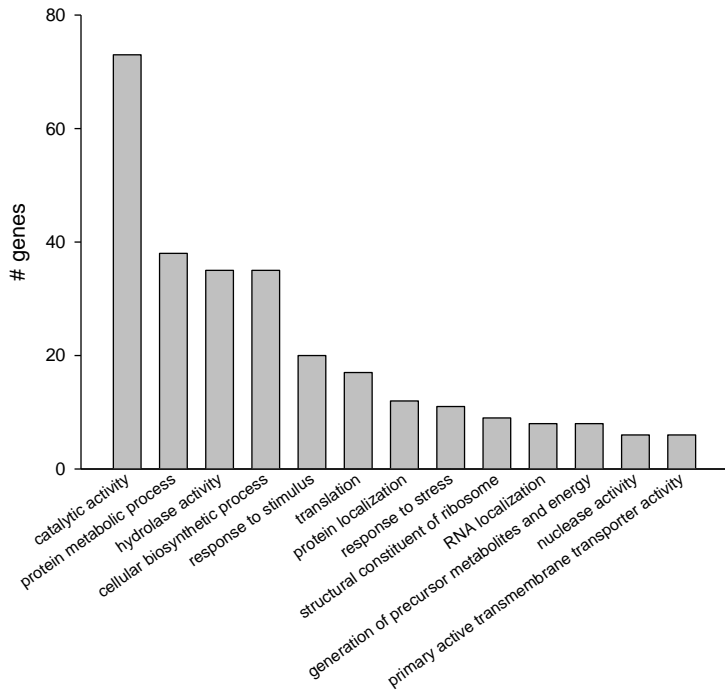**B**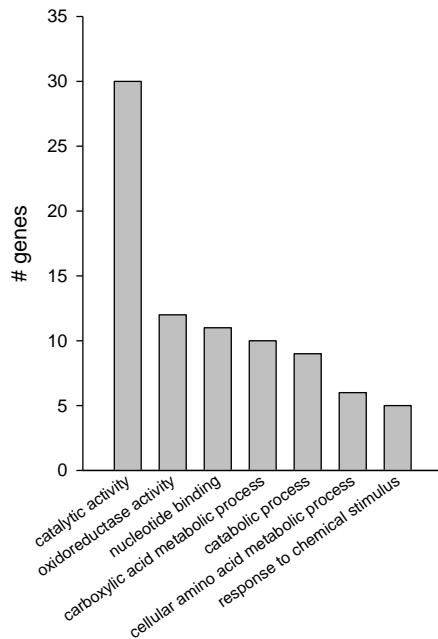

Supplement: Additional file 3 — Molecular function and biological process that were enriched in overlapping gene sets. A) Molecular function and biological process from genes that were upregulated in V-P+ but downregulated in V+P+. B) Molecular function and biological process from genes that were upregulated in V+P- but downregulated in V+P+. The number of genes differentially expressed in each pathway is shown. [file 1471-2164-12-496-S3.PDF]

relative mRNA expression level

*Sod*

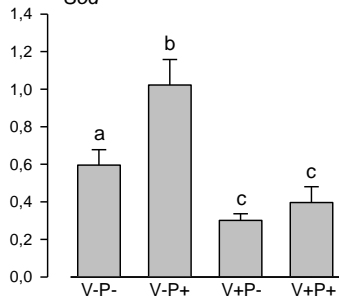

*Sod2*

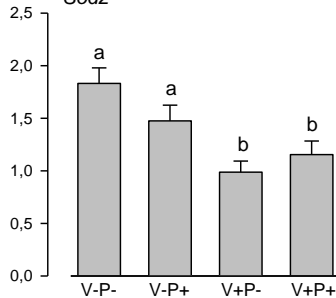

*Trxr-1*

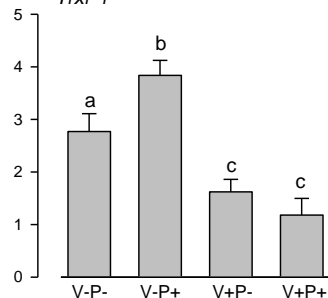

*PGRP-LC*

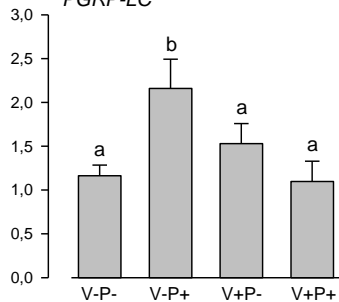

*defensin1*

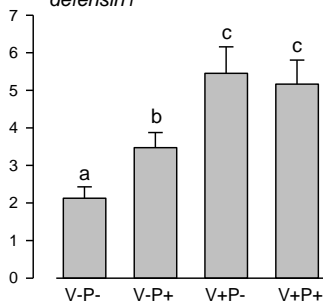

*Imd*

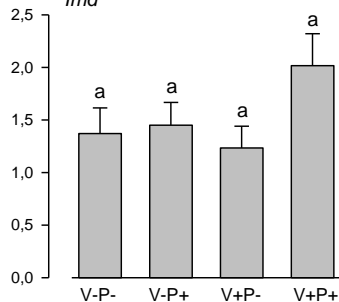

Supplement: Additional file 5 — Analysis with RT-PCR of selected immune and lifespan genes affected by pollen feeding or varroa parasitism. Expression values of selected lifespan (Sod, Sod2, Trxr-1) and immune genes (PGRP-LC, defensin1, Imd) differentially transcribed between the different treatments. RT-PCR data normalized to β-actin are shown. Means ± SE are shown for 8 pools of 3 bees per treatment. Different letters indicate significant differences detected by Mann-Whitney U tests. [file 1471-2164-12-496-S5.PDF]
