# Supplementary material for: Nutrigenomics in honey bees: digital gene expression analysis of pollen's nutritive effects on healthy and varroa-parasitized bees
Source: BMC Genomics. 2011 Oct 10;12:496. doi: 10.1186/1471-2164-12-496 (PMC3209670; doi:10.1186/1471-2164-12-496)
Supplement: Additional file 4 — Molecular pathways affected by pollen feeding or varroa parasitism. Pathways that were significantly enriched (P < 0.05) in the different gene sets are shown. The analysis was done with DAVID 6.7 bioinformatic resources. [file 1471-2164-12-496-S4.PDF]

Downregulated

Upregulated

V-P+/V-P-	V+P+/V+P-	V+P-/V-P-	V+P+/V-P+		
23	27	44		Ribosome	**Translation**
23	30			Spliceosome	
11	14	17		Aminoacyl-tRNA biosynthesis	
		19	28	Proteasome	**Protein and amino acid metabolism**
		14		Lysine degradation	
		10		Tryptophan metabolism	
17			22	Valine, leucine and isoleucine degradation	
9			14	beta-Alanine metabolism	
11				Arginine and proline metabolism	
6				Fatty acid elongation in mitochondria	**Lipid metabolism**
14			21	Fatty acid metabolism	
14				Glycolysis / Gluconeogenesis	**Energy metabolism**
12				Pyruvate metabolism	
13				Krebs cycle	
		42	59	Oxidative phosphorylation	
