# Supplementary material for: Nutrigenomics in honey bees: digital gene expression analysis of pollen's nutritive effects on healthy and varroa-parasitized bees
Source: BMC Genomics. 2011 Oct 10;12:496. doi: 10.1186/1471-2164-12-496 (PMC3209670; doi:10.1186/1471-2164-12-496)
Supplement: Additional file 6 — Sequences of qPCR primers. [file 1471-2164-12-496-S6.PDF]

| Gene name | Gene ID | | 5' to 3' | Reference |
|---|---|---|---|---|
| *Vg* | GB13999 | forward | TTGACCAAGACAAGCGGAACT | [1] |
| | | reverse | AAGGTTCGAATTAACGATGAA | |
| *mvl* | GB15139 | forward | CCTTGGTATAAAGATTATGACAGGAATATG | [2] |
| | | forward | CAAGAGCACTGTGAAGATACAAGTTATG | |
| *PPO* | GB18313 | reverse | AGATGGCATGCATTTGTTGA | [3] |
| | | forward | CCACGCTCGTCTTCTTTAGG | |
| *spz* | GB15688 | forward | TGCACAAATTGTTTTTCCTGA | [3] |
| | | reverse | GTCGTCCATGAAATCGATCC | |
| Sod | GB10133 | forward | GTCGTTCCGTGTAGTCGAGAA | [4] |
| | | forward | TCCTTTGACTTCACCCTGAAGA | |
| Sod2 | GB14346 | reverse | GGTGGTGGTCATTTGAATCATTC | [4] |
| | | forward | AAGAAGTGCAGCGTCTGGTTTAC | |
| Trxr-1 | GB14972 | forward | GCAGTGAATTTTGGTGCAAAAGT | [4] |
| | | reverse | CACCTAGACCCCAAGTGCTACC | |
| PGRP-LC | GB17188 | forward | TCCGTCAGCCGTAGTTTTTC | [3] |
| | | forward | CGTTTGTGCAAATCGAACAT | |
| defensin1 | GB19392 | reverse | TGCGCTGCTAACTGTCTCAG | [3] |
| | | forward | AATGGCACTTAACCGAAACG | |
| Imd | GB18606 | forward | TGTTAACGACCGATGCAAAA | [3] |
| | | reverse | CATCGCTCTTTTCGGATGTT | |
| human *β-actin* | | forward | GCTCGTCGTCGACAACGGCTC | SuperScript III kit |
| | | forward | CAAACATGATCTGGGTCATCTTCTC | Invitrogen |


1. Fischer P, Grozinger CM: **Pheromonal regulation of starvation resistance in honey bee workers (*Apis mellifera*)**. *Naturwissenschaften* 2008, **95**(8):723-729.
2. Ben-Shahar Y, Dudek NL, Robinson GE: **Phenotypic deconstruction reveals involvement of manganese transporter malvolio in honey bee division of labor**. *J Exp Biol* 2004, **207**(Pt 19):3281-3288.
3. Evans JD: **Beepath: an ordered quantitative-PCR array for exploring honey bee immunity and disease**. *J Invertebr Pathol* 2006, **93**(2):135-139.
4. Corona M, Hughes KA, Weaver DB, Robinson GE: **Gene expression patterns associated with queen honey bee longevity**. *Mech Ageing Dev* 2005, **126**(11):1230-1238.
